# Cardioprotective Effects of Sodium-Glucose Cotransporter Subtype Inhibition on Ischemic and Pharmacological Preconditioning

**DOI:** 10.7759/cureus.59757

**Published:** 2024-05-06

**Authors:** Takashi Egashira, Taiga Ichinomiya, Akihiro Yokoyama, Sojiro Matsumoto, Ushio Higashijima, Motohiro Sekino, Hiroaki Murata, Osamu Yoshitomi, Shuntaro Sato, Tetsuya Hara

**Affiliations:** 1 Department of Anesthesiology and Intensive Care Medicine, Nagasaki University Graduate School of Biomedical Sciences, Nagasaki, JPN; 2 Clinical Research Center, Nagasaki University Hospital, Nagasaki, JPN

**Keywords:** sodium-glucose cotransporter inhibitor, sodium-glucose cotransporter, type 2 diabetes, ischemia–reperfusion injury, ischemic preconditioning, cardioprotective effect

## Abstract

Background: Sodium-glucose cotransporter (SGLT) 2 inhibitors partially inhibit SGLT1 expression; however, whether a clinical dose of SGLT2 inhibitor abrogates ischemic preconditioning (IPC) is unknown, and the pharmacological cardioprotective effect under SGLT1 inhibition has not been examined. In this study, we investigated whether a clinical dose of tofogliflozin abrogates IPC and whether pharmacological preconditioning with olprinone has cardioprotective effects under SGLT1 inhibition.

Methods: Male Wistar rats were divided into seven groups (seven rats per group) and subjected to the following treatments before inducing ischemia/reperfusion (I/R; 30 minutes of coronary artery occlusion followed by 120 minutes of reperfusion): saline infusion control treatment (Con); ischemic preconditioning (IPC); IPC after phlorizin infusion (IPC+Phl); IPC after low-dose tofogliflozin infusion (IPC+L-Tof); IPC after high-dose tofogliflozin infusion (IPC+H-Tof); olprinone infusion (Olp); and Olp infusion after phlorizin infusion (Olp+Phl).

Results: The infarct size was significantly decreased in the IPC group, but not in the IPC+Phl group. In contrast, the infarct size decreased in the IPC+L-Tof and IPC+H-Tof groups. Additionally, Olp reduced the infarct size, and the effect was preserved in Olp+Phl groups. Phosphorylated AMP-activated protein kinase (AMPK) expression was lower in the IPC+Phl group compared to that in the IPC group.

Conclusion: The cardioprotective effect of IPC was attenuated by strong SGLT1 inhibition, but the effect was preserved under a clinical dose of highly selective SGLT2 inhibitor. Olprinone exerts a cardioprotective effect even under strong SGLT1 inhibition.

## Introduction

Ischemic heart diseases are the leading cause of death worldwide [1], and diabetes mellitus (DM) is an independent risk factor for cardiovascular (CV) events [2]. Furthermore, the relative risk for CV events in patients with DM is two to four times higher than that in patients without DM [3]. Sodium-glucose cotransporter (SGLT) 2 inhibitors are widely used anti-diabetic drugs that increase urinary glucose excretion [4]. In addition to their hypoglycemic effects, SGLT2 inhibitors have cardioprotective effects although the underlying mechanism is unclear. Empagliflozin, an SGLT2 inhibitor, significantly decreased CV-related death in the EMPA-REG OUTCOME trial [5]. Subsequently, a large-scale clinical trial [6] revealed that SGLT2 inhibitors can prevent CV events. Therefore, SGLT2 inhibitors have been increasingly prescribed to patients with DM with a risk of CV events.

Commercially available SGLT inhibitors are more selective for SGLT2 than for SGLT1; however, the degree of SGLT2 inhibition compared to that of SGLT1 varies widely among drugs, ranging from 155- to 2912-fold even for SGLT2 inhibitors, of which tofogliflozin is the most SGLT2-selective SGLT2 inhibitor [7]. The hypoglycemic effect of SGLT2 inhibitors diminishes over time owing to compensatory reabsorption through SGLT1 [8,9], prompting the recent development of sotagliflozin, a dual SGLT1/2 inhibitor exhibiting relatively low SGLT2 selectivity of 20-fold over SGLT1, to achieve improved glycemic control. Thus, the SGLT1 selectivity of SGLT2 inhibitors may be a crucial factor in future glycemic control.

SGLT1 is the only SGLT detected in the heart [10], and its expression is upregulated during pathological conditions, including ischemia and diabetes [11]. In myocardial ischemia-reperfusion (I/R) injury, the inhibition of SGLT1 expression suppresses glucose uptake into the myocardium via AMP-activated protein kinase (AMPK) and protein kinase B (Akt) activities and exacerbates infarction [12]. Additionally, ischemic preconditioning (IPC) upregulates SGLT1 expression via AMPK activity; however, the protective effect is diminished by phlorizin, an SGLT1 inhibitor [13]. Thus, inhibiting SGLT1 expression using SGLT2 and SGLT1/2 inhibitors could exacerbate myocardial I/R injury, even though it has recently been considered essential for improved glycemic control.

Olprinone, a phosphodiesterase III inhibitor, exerts cardioprotective effects via the PI3K-Akt pathway, independent of AMPK activity [14]. Because inhibiting cardioprotective effects using SGLT1 inhibitors may be associated with the inhibition of AMPK activity, pharmacological preconditioning with olprinone, which is independent of AMPK activity, may be effective even under SGLT1 inhibition. However, only a few studies investigated the effect of inhibiting SGLT1 expression using highly selective SGLT2 inhibitors on cardioprotective effects during I/R injury.

Therefore, the present study investigated whether the effect of SGLT1 inhibition by a highly selective SGLT2 inhibitor affects the cardioprotective effects of ischemic preconditioning and whether olprinone exerts a cardioprotective effect under SGLT1 expression inhibition, while also determining the molecular mechanism.

## Materials and methods

Animal care

The Institutional Animal Care and Use Committee of the Nagasaki University School of Medicine approved all animal experiments under approval number 1706121387. All methods were performed in accordance with the approved guidelines.

Drugs

Phlorizin was purchased from FUJIFILM Wako (Osaka, Japan), olprinone from Eisai (Tokyo, Japan), and 2,3,4-triphenyltetrazolium chloride (TTC) from Sigma-Aldrich (St. Louis, MO). Tofogliflozin was provided by Kowa Company, Ltd. (Nagoya, Japan).

General preparation

Male Wistar rats weighing 200-250 g and seven to eight weeks old were purchased from SLC (Hamamatsu, Japan). The rats were anesthetized with sodium pentobarbital (50 mg/kg intraperitoneal bolus followed by 20 mg/kg/h intravenous infusion). Catheters were inserted into the right jugular vein and the right carotid artery for fluid or drug administration and arterial blood pressure measurement. After the tracheostomy, a cannula was intubated, and pure oxygen was used for ventilation. The arterial blood pressure was continuously measured for hemodynamic monitoring with a transducer (blood pressure monitor link Sck-9082, Becton Dickinson, Tokyo, Japan) and an AP-641G blood pressure amplifier (Nihon-Koden, Tokyo, Japan) and shown on a polygraph system (Nihon-Koden).

I/R and ischemic preconditioning protocols

After the left thoracotomy, the left anterior descending (LAD) coronary artery was occluded for 30 minutes (ischemia), followed by 120 minutes of reperfusion. LAD occlusion was conducted using a 7-0 prolene suture for ligation, and reperfusion was performed by loosening the snare. IPC was achieved via four cycles of five-minute LAD occlusion followed by five-minute reperfusion.

Experimental protocol

In total, 97 rats were included in this experiment; 24 rats died owing to anesthesia overdose and LAD ligation. Ultimately, 49 rats were used in the experiment for infarct size determination, 12 for TUNEL (terminal deoxynucleotidyl transferase dUTP nick end labeling) staining, and 12 for western blotting.

**Figure 1:**
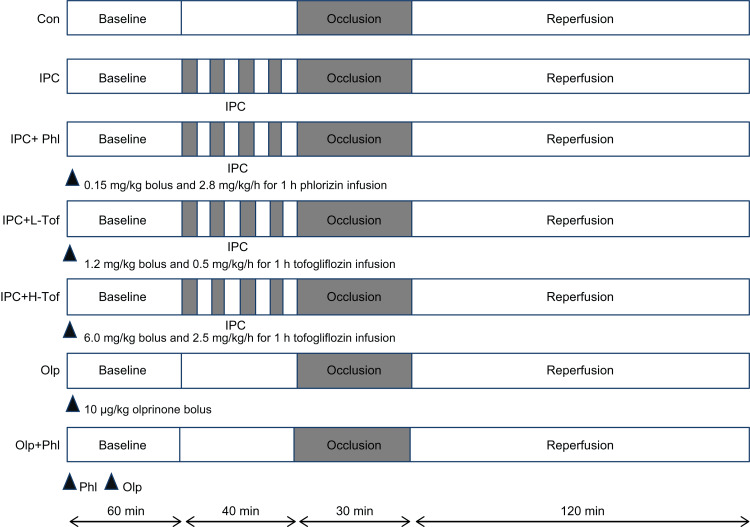
Schematic of the experimental timeline for each group. Con, control group; IPC, ischemic preconditioning group; Phl, IPC after phlorizin infusion group; Low-dose Tof, IPC after low-dose tofogliflozin (Tof) infusion; High-dose Tof, IPC after high-dose tofogliflozin infusion; Olp, olprinone infusion; Olp+Phl, Olp infusion after Phl infusion.

The experimental protocol is illustrated in Figure 1. Rats were divided into seven groups (seven rats per group) and subjected to the following treatments before inducing I/R: no treatment (Con); ischemic preconditioning (IPC); IPC after phlorizin infusion (IPC+Phl); IPC after low-dose tofogliflozin infusion (IPC+L-Tof); IPC after high-dose tofogliflozin infusion (IPC+H-Tof); olprinone infusion (Olp); and Olp infusion after phlorizin infusion (Olp+Phl).

The phlorizin and tofogliflozin doses were derived from a previous study on phlorizin and tofogliflozin plasma concentrations in rats [15]. One dose of tofogliflozin was administered at a blood concentration of 400 ng/mL (989898nM), the clinical concentration in humans [16], to mimic clinical settings. Additionally, based on the difference in the half maximal inhibitory concentration (IC50) values of tofogliflozin for SGLT2 inhibition (2.9 nM for humans and 14.5 nM for rats) [17], the other tofogliflozin dose was set at a five-fold higher dosage to achieve a similar SGLT2 inhibition in rats.

**Figure 2:**
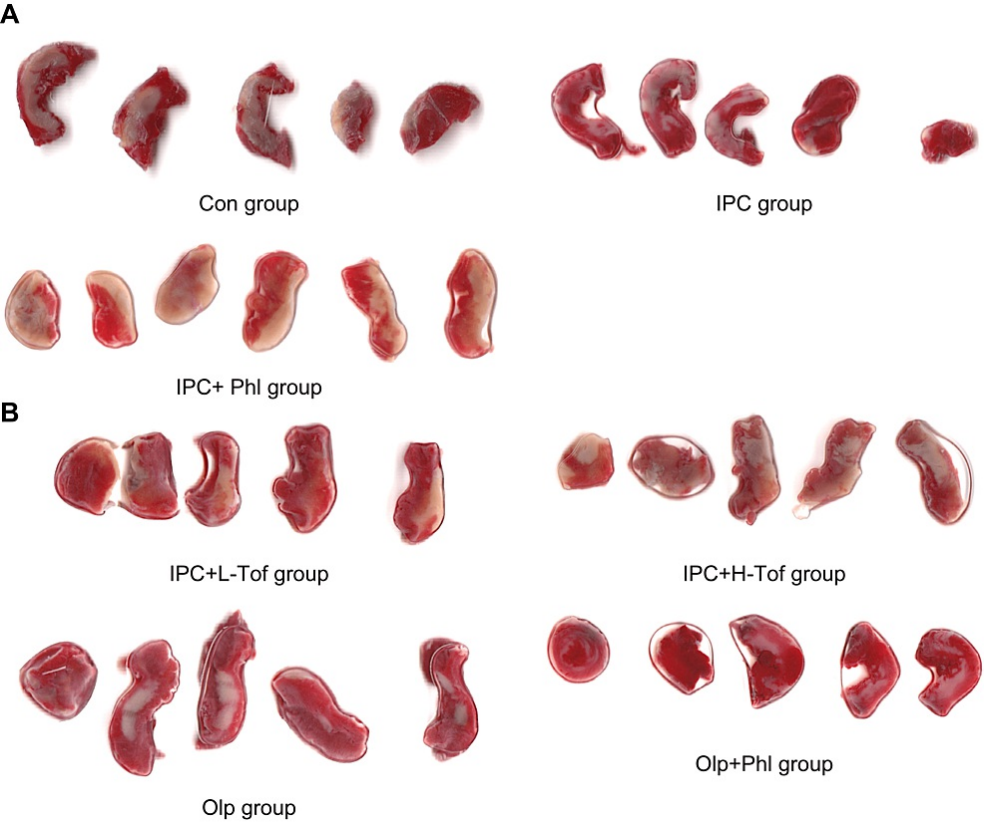
Myocardial section specimens in each group. Con, control; IPC, ischemic preconditioning; Phl, phlorizin; Tof, tofogliflozin; Olp, olprinone.

At the end of reperfusion, LAD occlusion was performed to identify the infarcted region, and patent blue dye was administered into the coronary artery. Stained areas with patent blue dye were identified as normal areas of the left ventricle (LV) and unstained areas were identified as the area at risk of ischemia (AAR). AAR was rapidly excised. The LV tissue was isolated and cut into approximately 10 cross-sectional pieces (Figure 2). AAR, the region of the myocardial bed supplied by the LAD coronary artery, was separated from the surrounding blue-stained LV normal area. The AAR and other LV areas were weighed to determine the AAR/LV ratio.

Determination of infarct size

At the end of the experiment, LAD was reoccluded and patent blue dye was administered into the right atrium to identify the ischemic region of the left ventricle. Stained areas with patent blue dye were normal, non-ischemic regions, and unstained areas were the areas at risk of ischemia (AAR). After the heart was excised, the LV tissue was isolated and cut into approximately 10 cross-sectional pieces. AAR, i.e. the region of the myocardial bed supplied by the LAD coronary artery, was separated from the surrounding, blue-stained LV normal area. The AAR and other LV areas were weighed to determine the AAR/LV ratio.

The AAR regions were incubated in 1% TTC in 0.1 M phosphate buffer adjusted to pH 7.4. TTC stains living tissue a red color, but the necrotic area is not stained and appears white within the AAR region (Figure 2). Each tissue slice was scanned at 1200 dpi with a commercial scanner (CanoScan LiDE400, Canon, Japan), and image analysis was performed by Image J bundled with Zulu OpenJDK 13.0.6 to derive the TTC negative area (necrotic tissue area)/AAR ratio.

The analysis of the infarct area was attempted by a different researcher than the one who performed the experiment, without being informed of which group was being analyzed.

Terminal deoxynucleotidyl transferase dUTP nick end labeling (TUNEL) assay

TUNEL staining was performed using an in situ apoptosis detection kit (MK500, Takara, Shiga, Japan) according to the manufacturer’s instructions. Peroxidase activity was visualized using diaminobenzene. The sections were counterstained with Mayer’s hematoxylin solution (Muto Pure Chemicals, Tokyo, Japan), dehydrated, and mounted with Malinol (Muto Pure Chemicals).

Western blotting

Phospho-AMPK and epidermal growth factor receptor (EGFR) levels were assessed using immunoblotting with specific isoform antibodies (cat# 2535 and 2646, Cell Signaling Technology, Danvers, MA). Sections of cardiac tissue were rapidly harvested from the infarcted area, flash-frozen in liquid nitrogen, and stored at -80°C. Each frozen specimen was crushed and homogenized in a cooled extraction buffer. After incubation at 4°C for 60 minutes, the samples were centrifuged at 1000 rpm and 4°C for 15 minutes to collect the middle layer, and centrifuged under the same conditions to collect the middle layer for the protein extract. Protein concentrations were determined using a bicinchoninic acid assay Protein Assay Kit (cat# 23225, Thermo Fisher Scientific, Waltham, MA) and standardized with a known amount of bovine serum albumin (BSA).

For western blot analysis, 40 µg of total protein was resolved in a 5-20% polyacrylamide gel (E-T520L cat# 2331830, ATTO, Daejeon, Korea) and transferred onto a polyvinylidene difluoride membrane. After incubation in a blocking solution of 0.05% Triton-Tris-buffered saline (TBS) containing 1% BSA, the membrane was immunoblotted with primary antibodies (1:1000 dilution) overnight at 4°C. The membrane was then washed thrice (five minutes each time) with 0.05% TBST (TBS with 0.05% Tween 20) and incubated with anti-rabbit IgG horseradish peroxidase (HRP)-linked secondary antibody (cat# 7074S, Cell Signaling Technology) for two hours at 24℃. The membranes were washed thrice, and proteins were detected using the Immobilon Western Chemiluminescent HRP Substrate. Signals were digitized and analyzed with ImageJ software bundled with Zulu OpenJDK 13.0.6 (NIH, Bethesda, MD).

Statistical analysis

The data are expressed as the central value (first to third quartile). The hemodynamic data, blood glucose concentrations, AAR value, and infarct size/AAR ratio were statistically analyzed using the Wilcoxon rank-sum test corrected using the Bonferroni method. Statistical analysis was performed using JMP® version 14 (SAS Institute, Cary, NC). A p-value of <0.05 was regarded as statistically significant.

## Results

**Figure 3:**
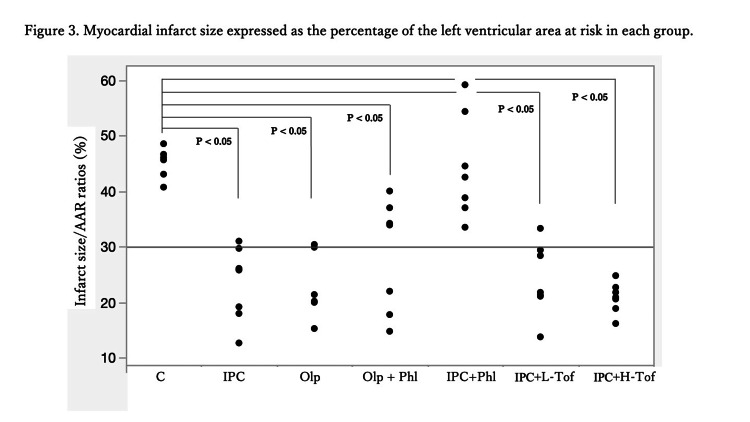
Myocardial infarct size expressed as the percentage of the left ventricular area at risk in each group. C, control; IPC, ischemic preconditioning; Phl, phlorizin; Tof, tofogliflozin; Olp, olprinone.

**Figure 4:**
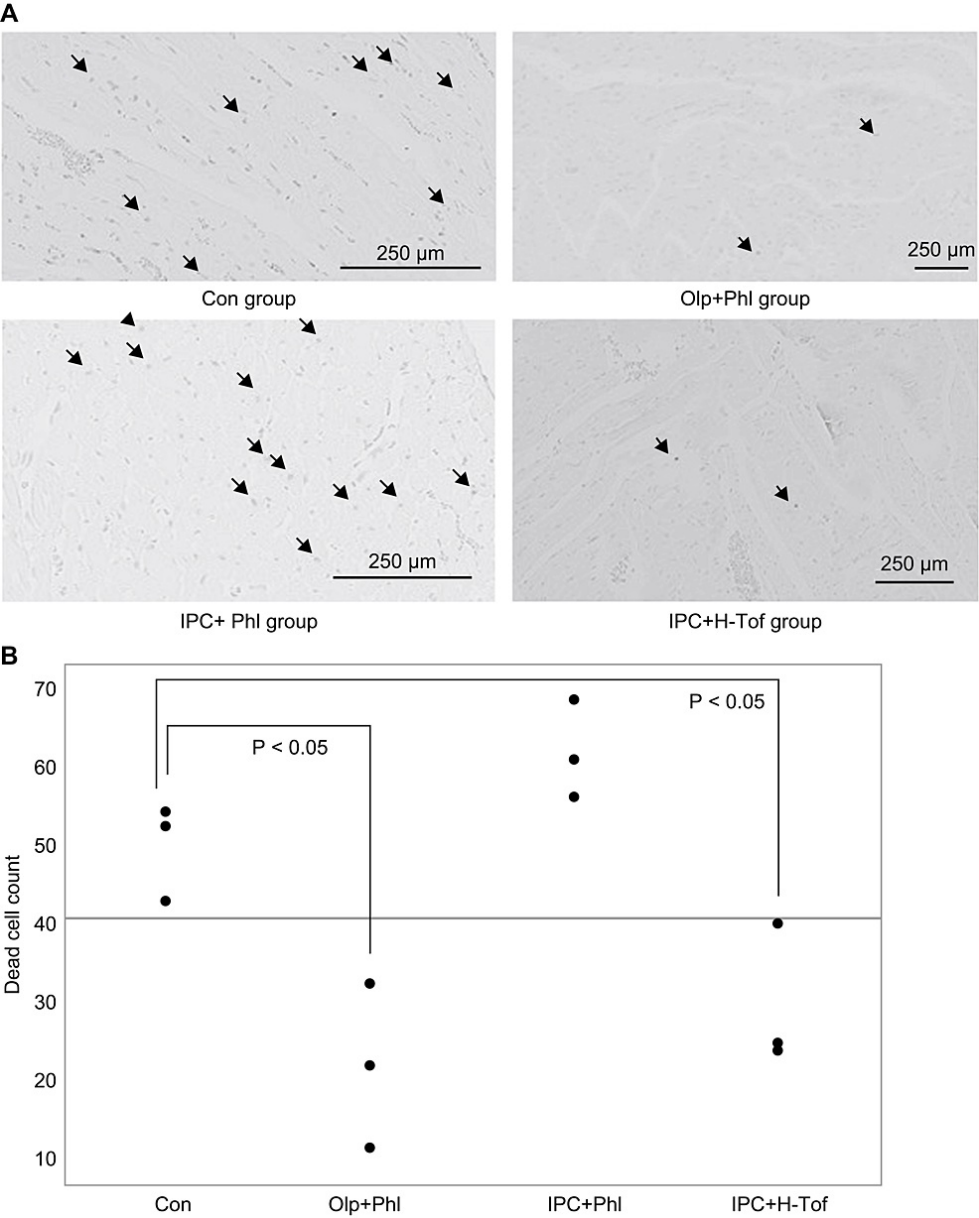
Apoptotic cells examined using TUNEL staining. Con, control; IPC, ischemic preconditioning; Phl, phlorizin; Tof, tofogliflozin; Olp, olprinone; TUNEL, terminal deoxynucleotidyl transferase dUTP nick end labeling.

**Figure 5:**
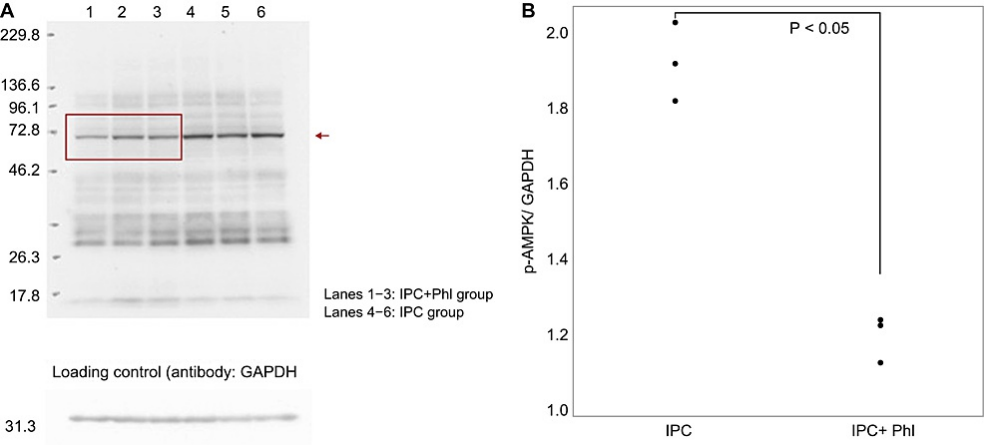
Phosphorylated AMP-activated protein kinase (p-AMPK) expression in cardiomyocytes in the Phl and IPC groups was detected using western blotting with anti-p-AMPK antibodies. Lanes 1–3: Phl group. Lanes 4–6: IPC group. Red arrow: p-AMPK. Loading control: detection with anti-glyceraldehyde 3-phosphate dehydrogenase (GAPDH) antibodies. IPC, ischemic preconditioning; Phl, phlorizin.

**Figure 6:**
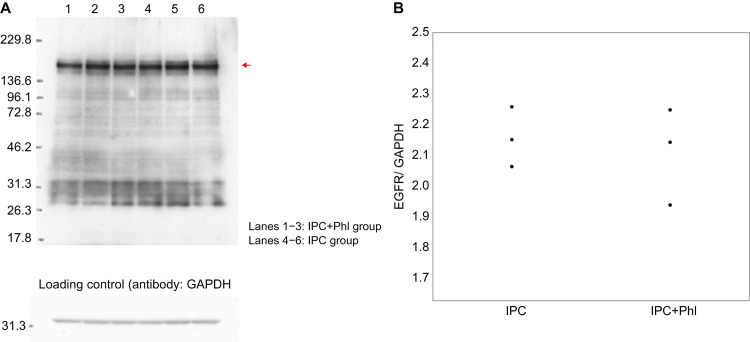
Epidermal growth factor receptor (EGFR) expression in cardiomyocytes in the Phl and IPC groups was detected using western blotting with anti-EGFR antibodies. Lanes 1–3: Phl group. Lanes 4–6: IPC group. Red arrow: EGFR. Loading control: detection with anti-glyceraldehyde 3-phosphate dehydrogenase (GAPDH) antibodies. IPC, ischemic preconditioning; Phl, phlorizin.

No significant differences in body weight were observed among the groups. The mean arterial pressure and heart rate did not significantly vary among the groups (Table 1). The plasma glucose level was measured before and 60 minutes after phlorizin or tofogliflozin infusion. No significant differences were observed in the blood glucose level among the groups (Table 2). The LV weight, AAR weight, and AAR/LV ratio were similar among the groups (Table 3). The infarct size/AAR ratio was significantly reduced in the IPC (25.8 (12.7-31) %), IPC+L-Tof (21.8 (13.8-33.3) %), IPC+H-Tof (20.9 (16.2-24.8) %), Olp+Phl (33.9 (14.8-40) %), and Olp (21.4 (15.3-30.5) %) groups compared with that in the Con group (46 (40.7-67.3)). The IPC+Phl group (42.5 (33.5-59.1)) and the Con group had similar infarct size/AAR ratios (Figures 2, 3). Apoptosis in myocardial tissue evaluated using TUNEL staining was measured in the Con, IPC+Phl, IPC+H-Tof, and Olp+Phl groups (Figure 4A). The apoptotic cell count was low in the IPC+H-Tof (24 (23.0-40.0) cells/field) and Olp+Phl (21 (10.0-32.0) cells/field) groups compared with that in the Con group (53 (43.0-55.0) cells/field). The apoptotic cell count of the IPC+Phl group (62 (57.0-70.0) cells/field) was similar to that of the Con group (Figures 4A, 4B). The protein expression of p-AMPK was significantly lower in the IPC+Phl group than in the IPC group (Figure 5); however, EGFR expression was similar in both groups (Figure 6).

**Table 1:** Systemic hemodynamics: mean blood pressure (mmHg). Central value (1st quartile-3rd quartile). Con, control; IPC, ischemic preconditioning; Phl, phlorizin; Tof, tofogliflozin; Olp, olprinone.

Group	Baseline	Intervention	Preocclusion	Occlusion, 15 minutes	Reperfusion, 1 hour	Reperfusion, 2 hours
Con	108, 91–130	105, 91–130	102, 83–120	102, 80–120	100, 75–111	93, 73–127
IPC	106, 89–125	104, 99–117	105, 90–128	95, 87–119	99, 82–117	101, 78–126
IPC+Phl	100, 100–135	108, 72–144	116, 78–131	94, 69–117	93, 70–113	95, 75–109
IPC+L-Tof	101, 78–124	98, 71–140	100, 66–128	96, 67–112	76, 62–133	100, 71–128
IPC+H-Tof	115, 100–142	121, 92–142	120, 84–145	116, 84–134	102, 83–145	91, 75–155
Olp	102, 74–124	100, 91–115	96, 84–119	104, 94–121	95, 83–120	91, 81–117
Olp+Phl	112, 77–122	111, 79–123	111, 83–123	115, 87–122	112, 83–129	110, 73–140

**Table 2:** Blood glucose concentrations (mg/dL). Central value (1st quartile-3rd quartile). IPC, ischemic preconditioning; Phl, phlorizin; Tof, tofogliflozin; Olp, olprinone.

Group	Baseline	Intervention, 60 minutes
IPC+Phl	109, 85–160	109, 89–114
IPC+L-Tof	114, 85–132	123, 102–156
IPC+H-Tof	120, 98–145	112, 96–154
Olp+Phl	112, 89–132	110, 93–135

**Table 3:** Left ventricular area at risk in each group. Central value (1st quartile-3rd quartile) Con, control; IPC, ischemic preconditioning; Phl, phlorizin; Tof, tofogliflozin; Olp, olprinone.

Group	Number	Area at risk/left ventricle (%)
Con	7	45.3, 42.9–50.3
IPC	7	44.3, 41.2–51.1
IPC+Phl	7	48.2, 43.4–49.3
IPC+L-Tof	7	45.3, 43.2–47.8
IPC+H-Tof	7	42.3, 40.5–48.3
Olp	7	46.3, 36.4–49.1
Olp+Phl	7	45.2, 41.0–53.3

## Discussion

Our findings demonstrated that phlorizin, an SGLT1 inhibitor, abolished the cardioprotective effects of IPC mediated by AMPK activation in vivo. In contrast, the effects of IPC were preserved under treatment with tofogliflozin, the most SGLT2-selective highly selective SGLT2 inhibitor. In addition, the cardioprotective effect of pharmacological preconditioning with olprinone was observed even under SGLT1 inhibition.

The predicted phlorizin concentration was approximately 3054 nmol/L in this study [15]. Previous I/R injury studies in rat models have used phlorizin at a concentration of approximately 0.1-10 mmol/L [13,18], considerably higher than the concentration used in this study. However, the IC50 value of phlorizin against rat SGLT1 is 970 nmol/L [17], well below the concentration used in this study. Thus, the phlorizin concentration used in this study is lower than that in previous studies but is still sufficient for SGLT inhibition and attenuation of the cardioprotective effect of IPC, and SGLT1 inhibition may suppress the cardioprotective effect of IPC.

The IC50 values of tofogliflozin for rat SGLT1 and SGLT2 are 8200 and 14.5 nmol/L, respectively, and the selectivity for SGLT2 over SGLT1 in rats is 560-fold lower than that in humans (2900-fold) [17]. Yoshii et al. compared the effect of tofogliflozin at different concentrations (5 and 50 mmol/L) during I/R in an isolated perfusion model. At 50 mmol/L, tofogliflozin significantly reduced cardiac function but not at 5 mmol/L [18]. In this study, the estimated blood concentration of tofogliflozin, adjusted to the concentration of human clinical dosage, was 400 ng/mL (approximately 989 nmol/L). This concentration was approximately 12% of the IC50 value of tofogliflozin for rat SGLT1 (8300 nmol/L) and did not attenuate the effect of IPC. Furthermore, the effect of IPC was not attenuated by tofogliflozin even at a five-fold concentration (approximately 60% of the IC50 value for rat SGLT1), selected based on the five-fold difference in IC50 for SGLT2 inhibition between humans and rats (2.9 vs. 14.5 nmol/L). Therefore, weak SGLT1 inhibition by tofogliflozin at the clinical dosage may not abrogate the cardioprotective effect of IPC.

SGLT1 plays a protective role in the myocardium during the acute phase of I/R injury to maintain cardiac energy metabolism, and its mechanism involves glucose uptake by increased SGLT1 expression via AMPK and Akt activities [13]. Additionally, AMPK activation may directly or indirectly upregulate SGLT1 expression [19], and AMPK upregulates SGLT1 expression through extracellular signal-regulated kinase (ERK) [20]. However, in this study, increased p-AMPK expression in IPC was suppressed by inhibiting SGLT1 expression with phlorizin. Thus, AMPK does not just upregulate SGLT1 expression but also may interact with SGLT1 during IPC. In contrast, EGFR expression, which increased in IPC in this study, was unaffected by phlorizin, whereas SGLT1 overexpression was attenuated by EGFR inhibition in a previous study [20]. EGFR is present in the cell membrane and regulates the activity of various downstream mediators and effectors such as AMPK, Akt, and ERK [19]. Therefore, SGLT1 inhibition by phlorizin may suppress the protective effect of IPC by acting downstream of EGFR.

AMPK may be a crucial mediator of abrogating cardioprotective effects during I/R injury under SGLT1 expression inhibition, and a cardioprotective approach independent of AMPK may be useful for this situation. Olprinone exerts a cardioprotective effect against I/R injury through the PI3K/Akt pathway, which is independent of AMPK and ERK signaling [14]. In agreement with the aforementioned study, this study showed that olprinone has a protective effect of pharmacological preconditioning even under SGLT inhibition. This is the first study to demonstrate effective pharmacological preconditioning under SGLT1 inhibition, and these results are important given the increasing focus on SGLT1 inhibition to improve glycemic control. However, the mechanism underlying the effect of olprinone should be investigated further.

Limitations

Cardioprotective Effect of SGLT2 Inhibitors

Although only SGLT1, and not SGLT2, is present in the myocardium [21], several studies have reported the cardioprotective effects of SGLT2 inhibitors on I/R injury [22-24], and the effect was independent of the hypoglycemic effect [24]. The mechanism of this protective effect is unclear; however, the potential mechanisms of the direct effect have been reported: inhibition of the Na+/H+ exchanger [25], a decreasing effect of intracellular Na+ and Ca2+ in cardiomyocytes [26], AMPK phosphorylation [27], and reducing oxidative stress [24]. However, Connelly et al. compared the effects of a dual SGLT1/2 inhibitor with a highly selective SGLT2 inhibitor on cardiac function after myocardial infarction; the dual SGLT1/2 inhibitor exacerbated cardiac dysfunction compared to the selective SGLT2 inhibitor [28]. Thus, SGLT2 inhibitors may have a cardioprotective effect in I/R injury, but their SGLT1 inhibitory effects are still important. In this study, tofogliflozin, a highly sensitive SGLT2 inhibitor, did not attenuate the cardioprotective effect of IPC, but further study is necessary to clarify whether tofogliflozin did not attenuate the cardioprotective effect of IPC owing to its weak SGLT1 inhibitory effect or the pharmacological protective effect of tofogliflozin itself.

SGLT1 Expression in Cardiomyocytes May Not Be Related to Glucose Uptake but I/R Injury

In a recent study, glucose uptake of cardiomyocytes was similar between SGLT1 knockdown mice and wild-type mice, and the inhibitory effect of glucose uptake by phlorizin was explained by glucose transporters (GLUTs) inhibition, independent of SGLT1 [29]. Thus, inhibition of the cardioprotective effect of IPC by phlorizin could also be independent of SGLT1. However, in another study, myocardial infarction after I/R injury decreased in cardiomyocyte-specific SGLT1-knockdown mice compared with that in wild-type controls, and AMPK up-regulated SGLT1 during ischemia [30]. In addition, membrane SGLT1 expression and p-AMPK in rat hearts were increased by IPC, but all beneficial effects of IPC were abolished by phlorizin [13]. Therefore, although SGLT1 inhibition of phlorizin may not be related to cardiomyocyte glucose uptake, SGLT1 still remains an important therapeutic target for I/R injury and the inhibitory effect of SGLT1 by phlorizin may be associated with the inhibition of cardioprotective effect by IPC. Further studies are necessary to determine the role of SGLT1 for cardioprotective effects during I/R injury.

## Conclusions

In this study, inhibition of SGLT1 abrogates preconditioning-induced cardioprotection against ischemia-reperfusion injury. The SGLT1 inhibitor abolished the cardioprotective effects of IPC; however, these effects were preserved under a highly selective SGLT2 inhibitor. Olprinone exerted the cardioprotective effect of pharmacological preconditioning even under SGLT1 inhibition, and AMPK inactivation by inhibiting SGLT1 expression may be a mechanism for attenuating the cardioprotective effects.
